# Investigation of the Lipid-Lowering Effect of Vitamin C Through GSK-3β/β-Catenin Signaling in Zebrafish

**DOI:** 10.3389/fphys.2018.01023

**Published:** 2018-08-14

**Authors:** Dongwu Liu, Hairui Yu, Qiuxiang Pang, Xiuzhen Zhang

**Affiliations:** ^1^Laboratory of Developmental and Evolutionary Biology, School of Life Sciences, Shandong University of Technology, Zibo, China; ^2^College of Biological and Agricultural Engineering, Weifang Bioengineering Technology Research Center, Weifang University, Weifang, China

**Keywords:** vitamin C, GSK-3β, β-catenin, C/EBPa, lipid-lowering effect, zebrafish

## Abstract

Vitamin C (VC) is an essential nutrient for most fish species because of the absence of L-gulonolactone oxidase in the bodies of fish. VC plays a significant role in maintaining the physiological functions and in improving the growth performance, immunity, and survival of fish. In this study, zebrafish (*Danio rerio*) were treated with 8.2, 509.6, and 1007.5 mg/kg VC diets for 2 weeks, and the muscle samples were collected for gene expression analysis and biochemical index analysis. The results indicated that 509.6 and 1007.5 mg/kg VC diets inhibited glycogen synthase kinase-3β (GSK-3β) expression and induced the expression of β-catenin in the muscle of zebrafish. The mRNA expression of CCAAT/enhancer-binding protein α (C/EBPα) and fatty acid synthase (FAS), FAS activity, and the content of glycerol and triglyceride (TG) were decreased in the muscle by 509.6 and 1007.5 mg/kg VC diets. In addition, GSK-3β RNA interference was observed in zebrafish fed with 8.2 and 1007.5 mg/kg VC diets. It was found that GSK-3β RNA interference induced the mRNA expression of β-catenin but decreased the mRNA expression of C/EBPα and FAS, FAS activity, as well as the content of glycerol and TG in the muscle of zebrafish. In ZF4 cells, the mRNA expression of GSK-3β, C/EBPα, and FAS was decreased, but β-catenin expression was increased by 0.1 and 0.5 mmol/L VC treatments *in vitro*. The glycerol and TG content, and FAS activity in ZF4 cells were decreased by 0.1 and 0.5 mmol/L VC treatments. Moreover, the result of western blot indicated that the protein expression level of GSK-3β was significantly decreased and that of β-catenin was significantly increased in ZF4 cells treated with 0.1 and 0.5 mmol/L VC. The results from *in vivo* and *in vitro* studies corroborated that VC exerted the lipid-lowering effect through GSK-3β/β-catenin signaling in zebrafish.

## Introduction

Vitamin (VC) is an essential nutrient for most fish species because of the absence of L-gulonolactone oxidase in the fish body (Fracalossi et al., 2001). In the previous studies, it was found that the minimum requirement of VC was 10∼25 mg/kg diet for rainbow trout (*Oncorhynchus mykiss*) (Cho and Cowey, 1993), channel catfish (*Ictalurus punctatus*) (Mustin and Lovell, 1992), hybrid tilapia (*Oreochromis niloticus* ×*O. aureus*) (Shiau and Hsu, 1999), and hybrid striped bass (*Morone chrysops* ×*M. saxatilis*) (Sealey and Gatlin, 1999). For large yellow croaker (*Pseudosciaena crocea*), the VC requirement was 28.2 mg/kg diet based on the survival, whereas it was 87.0 mg/kg diet based on the VC content in liver (Ai et al., 2006). Dietary VC requirement of juvenile largemouth bass (*Micropterus salmoides*) based on growth was 102.6∼109.5 mg/kg (Chen et al., 2015), and it was 410.8 mg/kg diet based on the VC content in liver for Japanese eel (*Anguilla japonica*) (Shahkar et al., 2015).

As an essential nutrient for fish species, VC plays a significant role in maintaining the normal physiological functions (Khan et al., 2017). It has been observed that VC decreases the oxidative damage from lipid peroxidation and improves the growth performance, immunity, and survival of fish (Madhuban and Kaviraj, 2009; Norouzitallab et al., 2009; Gao et al., 2013; Wan et al., 2014; Barros et al., 2014). In mammals, the dietary VC is negatively associated with the occurrence of obesity and regulates adipocyte lipolysis, hyperglycemia, and glycosylation (Garcia-diaz et al., 2014; Christie-David et al., 2015; Ellulu et al., 2015; Park et al., 2015; Wilson et al., 2017). VC improves the plasma lipid profile, inhibits the lipid oxidation of high-density lipoprotein cholesterol (HDL-C), and prevents atherogenic modification of low-density lipoprotein cholesterol (LDL-C) (Karakilcik et al., 2014; Ashor et al., 2016). Since VC decreases the total serum cholesterol, LDL-C, and very-low-density lipoprotein (VLDL) and reduces the risk of coronary heart disease, it has been used to treat atherosclerosis by controlling cholesterol levels (Ashor et al., 2014; Zhang et al., 2014; Kobylecki et al., 2015; Polidori et al., 2015).

In the canonical Wnt signaling pathway, without Wnt ligands, β-catenin is continuously ubiquitinylated for degradation through glycogen synthase kinase-3β (GSK-3β). Upon Wnt ligand binding to receptors at membrane, a cascade of events impedes GSK-3β activity (Taelman et al., 2010; Arrázola et al., 2015). The ensuing accumulation and nuclear translocation of β-catenin leads to the transcription of a genetic program specific for the temporal, spatial, and tissue contexts (Valenta et al., 2012; Acebron and Niehrs, 2016). As a serine/threonine kinase, GSK-3β is involved in regulating numerous signaling pathways. The deregulation of GSK-3β is related to the development of diabetes, neurodegenerative disease, and bipolar disorder (Beurel et al., 2015; Maqbool et al., 2016). GSK-3β plays a significant role in regulating the canonical Wnt/β-catenin signaling pathway (Dema et al., 2016; Jain et al., 2017). Moreover, it has been found that Wnt/β-catenin signaling participates in regulating the cholesterol metabolism and triglyceride (TG) accumulation and affects the hepatic glucose, glycogen, and lipid metabolism (Chen et al., 2014; Kim et al., 2015; Popov et al., 2016; Liu et al., 2016b).

In fish species, muscle is one of the main sites for lipid storage (Sheridan, 1988). Adipocytes are present in the myoseptum of muscle, and lipid storage in the myoseptum is 40% of white muscle lipid content (Zhou et al., 1995, 1996; Nanton et al., 2007). It has been found that VC influenced lipid composition and peroxidation in the hepatopancreas and muscle of common carp (*Cyprinus carpio*) (Hwang and Lin, 2002). In our previous study, Wnt/β-catenin signaling participated in regulating lipogenesis in juvenile turbot (*Scophthalmus maximus*) (Liu et al., 2016b). However, the regulatory mechanism of VC on lipid deposition remains largely unknown in fish species. Thus, it is interesting to investigate whether VC affects lipid profile via GSK-3β/β-catenin signaling. In this study, the lipid-lowering effect of VC through GSK-3β/β-catenin signaling was investigated in zebrafish (*Danio rerio*) by an *in vivo* and *in vitro* study.

## Materials and Methods

### Diets for VC Experiment *in vivo*

The basal diet was formulated according to **Table 1**. Then the different contents of L-Ascorbate-2-phosphate (VC, Hangzhou Haijin Biology Technology Co., Ltd.) were added to the basal diets. The content of VC in the diet was measured with high efficiency liquid chromatography, and 8.2, 509.6, and 1007.5 mg/kg VC was detected in three diets, respectively.

**Table 1:** Composition and proximate analysis of the experimental diets.

Items	VC level (g/kg diet)		
	0	0.5	1.0
**Ingredients (g/kg diet)**			
Casein	420	420	420
Gelatin	105	105	105
Dextrin	190	190	190
Lard oil	83	83	83
Linseed oil	17	17	17
Cellulose	100	99.5	99
Sodium carboxymethylcellulose	20	20	20
Vitamin premix^1^	10	10	10
Mineral premix^2^	40	40	40
Ca_2_(H_2_PO_4_)_2_	10	10	10
Choline chloride	5	5	5
VC	0	0.5	1.0
total	1000	1000	1000
**Proximate composition**			
VC (mg/kg diet)	8.2	509.6	1007.5
Moisture (g/kg diet)	96.3	98.8	97.7
Crude protein (g/kg diet)	482.6	485.2	486.8
Crude lipid (g/kg diet)	106.4	105.9	108.5
Crude ash (g/kg diet)	63.1	65.4	64.6

*(1) Vitamin premix contained (mg/g mixer) thiamin hydrochloride, 5 mg; riboflavin, 5 mg; calcium pantothenate, 10 mg; nicotic acid, 6.05 mg; pyridoxine hydrochloride, 4 mg; folic acid, 1.5 mg; inositol, 200 mg; menadione, 4 mg; alpha-tocopherol acetate, 50 mg; retinyl acetate, 60 mg; biotin, 0.6 mg. All ingredients were diluted with alpha-cellulose to 1 g. (2) Mineral premix contained (g/kg diet) calcium biphosphate, 13.58 g; calcium lactate, 32.7 g; FeSO_4_⋅6H_2_O, 2.97 g; magnesium sulfate, 13.7 g; potassium phosphate dibasic, 23.98 g; sodium biphosphate, 8.72 g; sodium chloride, 4.35 g; AlCl_3_⋅6H_2_O, 0.015 g; KI, 0.015 g; CuCl_2_, 0.01 g; MnSO_4_⋅H_2_O, 0.08 g; CoCl_2_⋅6H_2_O, 0.1 g; ZnSO_4_⋅7H_2_O, 0.3 g.*

### Experimental Animals and VC Experiment *in vivo*

Zebrafish (AB strain) were maintained at a light:dark photoperiod (12-h:12-h) at 28°C in the dechlorinated water in the flow-through tanks. The zebrafish were, on average, ∼3.4 cm in length and 6 months of age. Twelve glass tanks were randomly distributed with 72 male zebrafish. Animals were fed twice daily with a commercial diet (Sanyou Beautification Feed Tech Co., Ltd, China) and were allowed to get acclimated for 15 days. Subsequently, the diet containing each level of VC was randomly assigned to four tanks. Fish were fed to apparent satiation twice daily (08:00 and 18:00).

Two weeks later, fish were anesthetized with 0.1 g/L MS222, and four independent white muscle samples were collected from four tanks, of which each sample was pooled from the dorsum of three fish. Four muscle samples were frozen in liquid nitrogen and stored at -80°C for molecular biology analysis. In addition, the muscle from the other three fish were sampled and pooled for the biochemical analysis. Four samples were homogenized in cold saline with a glass homogenizer and centrifuged at 4000 ×*g* for 15 min at 4°C, and the supernatants were collected for biochemical analysis. All animal experiments were approved by Shandong University of China’s Institutional Animal Care Committee in accordance with the Guidelines for Proper Conduct of Animal Experiments (Science Council of China).

### GSK-3β RNA Interference *in vivo*

Firstly, the open reading frame (ORF) of GSK-3β gene (NM_131381.1) was cloned with the primers (F1: 5′-CTG GTG AGC AGT AGG GTG-3′; R1: 5′-CGG ATT CGT TCA AGA CAA). According to the method of Arockiaraj et al. (2014), the double-stranded RNA (dsRNA) was synthesized with the primers containing the T7 promoter RNA polymerase recognition sequence at the 5′ end (F2: 5′-GAT CAC TAA TAC GAC TCA CTA TAG GGC GGC ATT CGG CAG CAT GAA AG-3′; R2: 5′-GAT CAC TAA TAC GAC TCA CTA TAG GGG CAC GGC TGT GTC TGG GTC CA-3′). The reactions of *in vitro* transcription were performed in water at 37°C in a solution containing T7 RNA polymerase (Takara), 100 mM DTT, RNase Inhibitor, 5 × transcription buffer, 2.5 mM rNTP, and the template. A UV spectrophotometer (Nano View) was used to detect the concentration of dsRNA. Then 72 male zebrafish were randomly distributed into 12 glass tanks, which included four groups and each group included three tanks: group 1 (DEPC water + 8.2 mg/kg VC), group 2 (RNAi + 8.2 mg/kg VC), group 3 (DEPC water + 1007.5 mg/kg VC), and group 4 (RNAi + 1007.5 mg/kg VC). For the groups of GSK-3β RNA interference (group 2 and group 4), each fish received intraperitoneal injection of 25 μL dsRNA (8ng/μL). For the control groups (group 1 and group 3), each fish was injected with 25 μL DEPC water. The diet containing 8.2 mg/kg VC was fed to the fish in group 1 and group 2, and the diet containing 1007.5 mg/kg VC was fed to fish in group 3 and group 4, respectively. Seven days later, fish were sampled for VC treatment *in vivo*.

### ZF4 Cell Culture and VC Treatment *in vitro*

ZF4 cells of zebrafish were purchased from China Zebrafish Resource Center (CZRC) (Wuhan, China), and maintained in DMEM:F12 (Gibco) supplemented with 10% fetal bovine serum at 28°C (5% CO_2_). ZF4 cells were cultured in 0, 0.1, and 0.5 mmol/L VC diluted in the basal culture medium for 48 h. Then cells were collected for gene expression analysis and biochemical analysis. The experiment was repeated three times.

### Assay the Content of Glycerol and TG, and the Activity of Fatty Acid Synthase (FAS), and the Protein Concentration

Muscle was homogenized and ZF4 cell samples were treated with 0.2% Triton X-100. The supernatants were collected for biochemical analysis after centrifugation at 4000 ×*g* for 10 min. According to the method of Liu et al. (2016a,b), the content of glycerol and TG, FAS activity, and the protein concentration were assayed with the glycerol, TG, FAS activity kits purchased from Nanjing Jiancheng Bioengineering Institute (Nanjing, China).

### RNA Extraction and Real-Time Quantitative Polymerase Chain Reaction

Total RNA was extracted from muscle or ZF4 cells using Trizol reagent (Invitrogen, United States) and transcribed to cDNA by PrimeScript^TM^ RT Reagent Kit (Takara, Japan). The primer sequences for GSK-3β, β-catenin, FAS, CCAAT/enhancer-binding protein α (C/EBPα), and reference gene (β-actin) (Teng et al., 2014) were listed in **Table 2**. A quantitative thermal cycle (ROCHE, Lightcycler96, Switzerland) and SYBR^®^ Premix Ex Taq^TM^ II (Takara, Japan) were used to carry out real-time PCR. The real-time PCR program was set as follows: 50°C for 2 min, 95°C for 10 min, followed by 40 cycles of 95°C for 15 s, and 60°C for 1 min. The amplification efficiency was detected, and the 2^-ΔΔC_T_^ method was employed to analyze the differences of relative gene expression in each sample by using β-actin as the internal reference gene (Livak and Schmittgen, 2001).

**Table 2:** Real-time quantitative PCR primers for the genes of zebrafish.

Target gene	Forward (5′-3′)	Reverse (5′-3′)	Size (bp)	GenBank
GSK-3β	TCTGCTCACCGTTTCCTTTC	CTCCGACCCACTTAACTCCA	115	NM_131381.1
β-catenin	GGAGCTCACCAGCTCTCTGT	TAGCTTGGGTCGTCCTGTCT	120	NM_001001889.1
C/EBPα	CACAACAGCTCCAAGCAAGA	AATCCATGTAGCCGTTCAGG	121	BC063934.1
FAS	ACAATGCTGGTGACAGTGGA	TACGTGTGGGCAGTCTCAAG	120	XM_009306806.2
β-actin	CCGTGACATCAAGGAGAAGC	TACCGCAAGATTCCATACCC	194	AF057040.1

### Immunofluorescent Microscopy of ZF4 Cells

ZF4 cells were cultured in basal culture medium containing 0, 0.1, and 0.5 mmol/L VC for 48 h. After the cells were fixed with paraformaldehyde and permeabilized with Triton X-100, cells were incubated with antibodies, anti-GSK-3β (Cat. No. 12456, Cell signaling, 1:200), and anti-β-catenin (Cat. No. 8480, Cell signaling, 1:200), respectively. Then cells were stained with the fluorescein isothiocyanate (FITC) dye secondary antibody (Santa-Cruz, 1:400). The negative controls with only secondary antibody were also performed. Finally, the samples were incubated with 5 μM DRAQ5™ (Cell signaling) nuclear stain, and a laser scanning confocal microscope (Leica TCS SP2, Germany) was used to detect the immunofluorescence.

### Western Blot Analysis of ZF4 Cells

ZF4 cells were cultured in basal culture medium containing 0, 0.1, and 0.5 mmol/L VC for 48 h. The ZF4 cell samples were prepared using the method described by Tian et al. (2018). The membranes were probed with primary antibody and the appropriate horseradish peroxidase (HRP)-conjugated secondary antibody. Antibodies directed against β-catenin (Cat. No. 8480, 1:1000), GSK-3β (Cat. No. 12456, 1:1000), Phospho-GSK-3β (Ser9) (Cat. No. 9323, 1:1000), and Phospho-β-Catenin (Ser675) (Cat. No. 4176, 1:1000) were purchased from Cell Signaling Technology Inc. β-actin (sc-1615, 1:10000) was purchased from Santa-Cruz Inc. Densitometry analyses were performed with the Image J software (National Institutes of Health).

### Statistical Analysis

Data were expressed as mean values ± standard error of mean (s.e.m). The statistical analyses were conducted using SPSS 16.0 (SPSS Inc, Chicago, IL, United States). The homogeneity of variances among groups and normality were tested, and the differences among samples were compared using one-way analysis of variance (ANOVA) followed by Tukey’s test. In addition, for GSK-3β RNA interference experiment, two-way ANOVA followed by the appropriate *post hoc* test was carried out to calculate the interaction of the two factors, “RNAi” and “VC content”, on the levels of gene expression and biochemical index. *P* < 0.05 was considered statistically significant.

## Results

### Effect of VC on the Levels of Gene Expression in the Muscle

**FIGURE 1:**
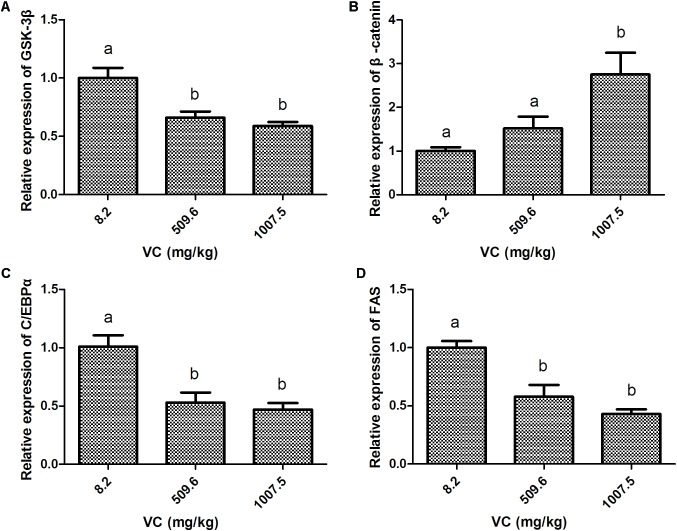
Effect of VC on the mRNA expression in the muscle of zebrafish. **(A)**: GSK-3β; **(B)**: β-catenin; **(C)**: C/EBPα; **(D)**: FAS. Values are expressed as means ± s.e.m. (*n* = 4). Statistically significant differences are denoted by different letters (*P* < 0.05).

Compared with the control group, the mRNA expression of GSK-3β in the muscle of zebrafish was significantly inhibited by 509.6 and 1007.5 mg/kg VC treatments for 2 weeks (*P* < 0.05) (**Figure 1A**). However, the mRNA expression of β-catenin was significantly induced by 1007.5 mg/kg VC treatment (*P* < 0.05) (**Figure 1B**). The mRNA expression of β-catenin in 509.6 mg/kg VC group was higher than the control but no significant difference was observed (**Figure 1B**). In addition, the mRNA expression of C/EBPα and FAS in the muscle of zebrafish was significantly decreased by 509.6 and 1007.5 mg/kg VC treatments (*P* < 0.05) (**Figures 1C,D**). No significant difference in the mRNA expression of C/EBPα and FAS was observed between the diets of 509.6 and 1007.5 mg/kg VC (**Figures 1C,D**).

### Effect of VC on FAS Activity, the Content of Glycerol and TG in the Muscle

**FIGURE 2:**
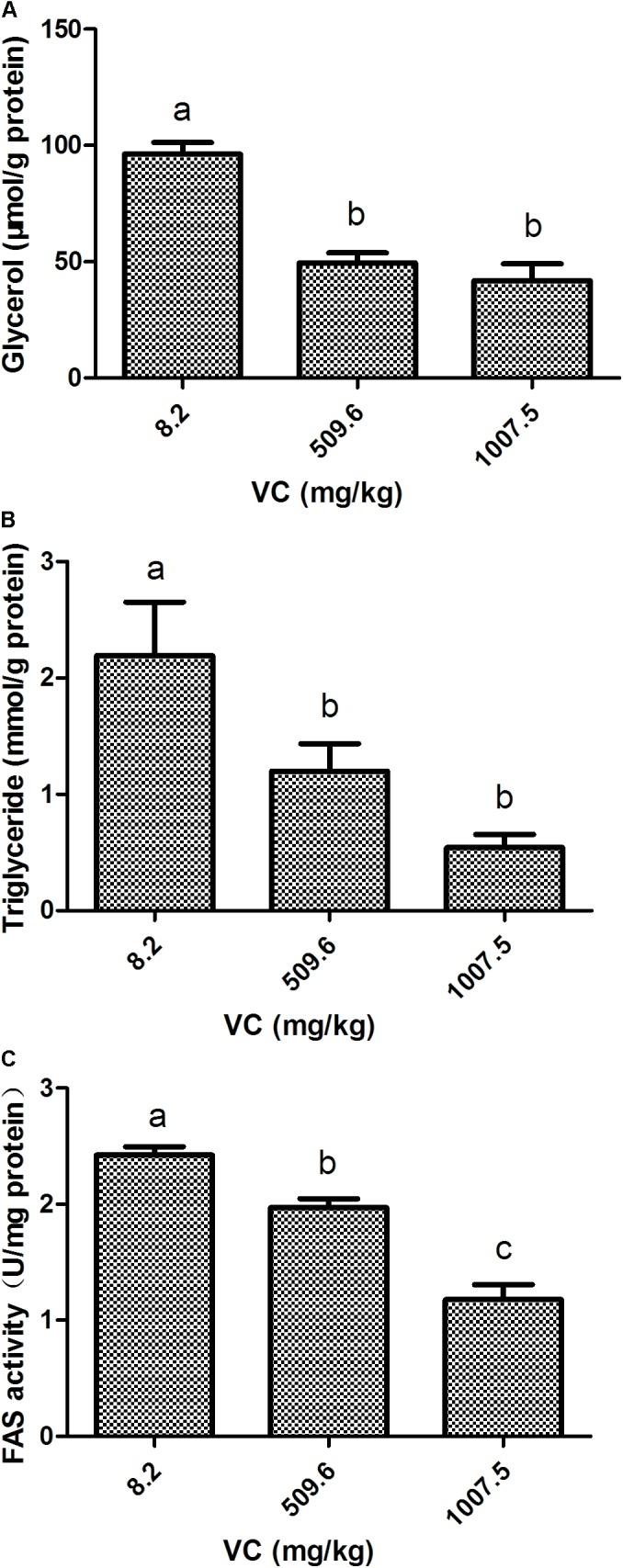
Effect of VC on glycerol, TG, and FAS activity in the muscle of zebrafish. **(A)**: glycerol content; **(B)**: TG content; **(C)**: FAS activity. Values are expressed as means ± s.e.m. (*n* = 4). Statistically significant differences are denoted by different letters (*P* < 0.05).

Compared with the control, the content of glycerol and TG in the muscle was significantly decreased by 509.6 and 1007.5 mg/kg VC treatments (*P* < 0.05) (**Figures 2A,B**). However, there was no significant difference between 509.6 and 1007.5 mg/kg VC treatments (**Figures 2A,B**). The activity of FAS in the muscle was significantly decreased by 509.6 and 1007.5 mg/kg VC treatments (*P* < 0.05), and there was significant difference between these two groups (*P* < 0.05) (**Figure 2C**).

### Effect of GSK-3β RNA Interference on the Levels of Gene Expression in the Muscle

**FIGURE 3:**
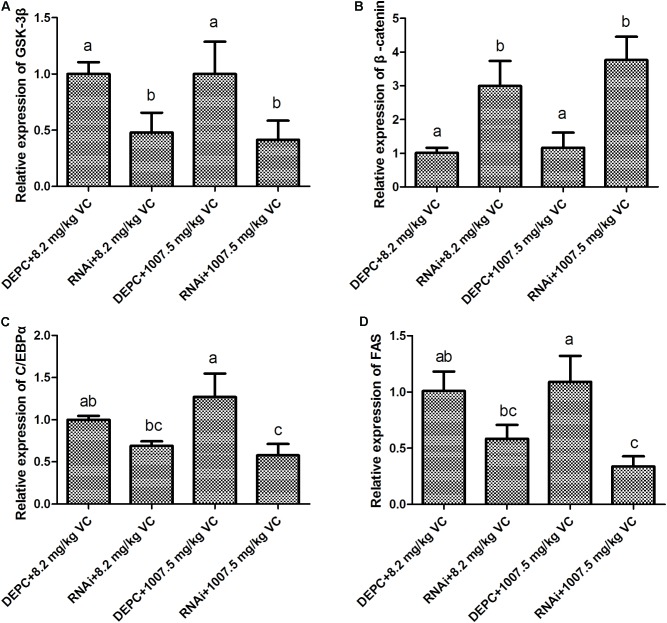
Effect of GSK-3β RNA interference on the mRNA expression in the muscle of zebrafish. **(A)**: GSK-3β; **(B)**: β-catenin; **(C)**: C/EBPα; **(D)**: FAS. Values are expressed as means ± s.e.m. (*n* = 3). Statistically significant differences are denoted by different letters (*P* < 0.05).

Compared with the control, the mRNA expression of GSK-3β was significantly decreased by GSK-3β RNA interference in the muscle of zebrafish fed with 8.2 and 1007.5 mg/kg VC diets a week later (*P* < 0.05) (**Figure 3A**). However, the mRNA expression of β-catenin was significantly increased by GSK-3β interference in the zebrafish fed with 8.2 and 1007.5 mg/kg VC diets (*P* < 0.05) (**Figure 3B**). Furthermore, the mRNA expression of C/EBPα and FAS was significantly decreased by GSK-3β interference fed with 1007.5 mg/kg VC diet (*P* < 0.05) (**Figures 3C,D**). Two-way ANOVA revealed that the factor RNAi had significant effect on the expression levels of GSK-3β, β-catenin, C/EBPα, and FAS (*P* < 0.05) (**Supplementary Table 1**). However, no significant difference was found on the factor VC content and the interaction factor (RNAi and VC content) (**Supplementary Table 1**).

### Effect of GSK-3β RNA Interference on FAS Activity and the Content of Glycerol and TG in the Muscle

**FIGURE 4:**
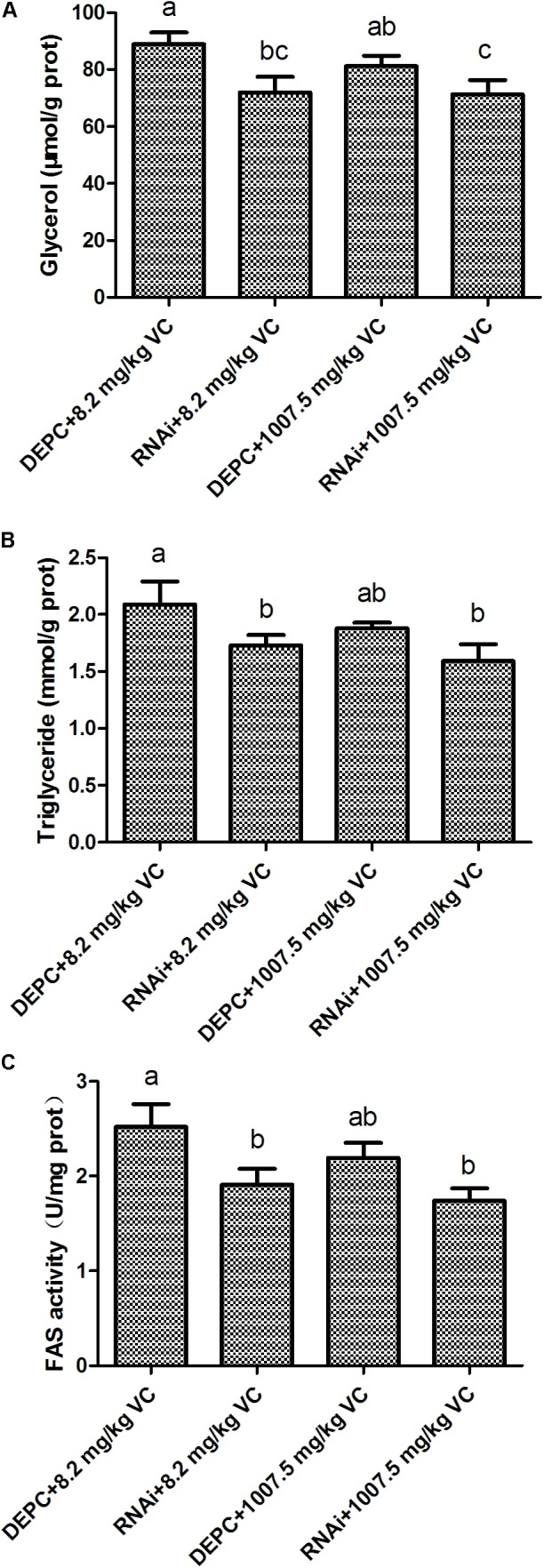
Effect of GSK-3β RNA interference on glycerol, TG, and FAS activity in the muscle of zebrafish. **(A)**: glycerol content; **(B)**: TG content; **(C)**: FAS activity. Values are expressed as means ± s.e.m. (*n* = 3). Statistically significant differences are denoted by different letters (*P* < 0.05).

The content of glycerol and TG was significantly decreased by GSK-3β RNA interference in the muscle of zebrafish fed with 8.2 and 1007.5 mg/kg VC diets (*P* < 0.05) (**Figures 4A,B**), and GSK-3β interference significantly affected the activity of FAS in the muscle of zebrafish fed with 8.2 and 1007.5 mg/kg VC diets (*P* < 0.05) (**Figure 4C**). Two-way ANOVA analysis indicated that the factor RNAi had significant effect on FAS activity and the content of glycerol and TG (*P* < 0.05), but no significant difference was found on the interaction factor (RNAi and VC content) (**Supplementary Table 2**).

### Effect of VC on the Levels of Gene Expression in the ZF4 Cells

**FIGURE 5:**
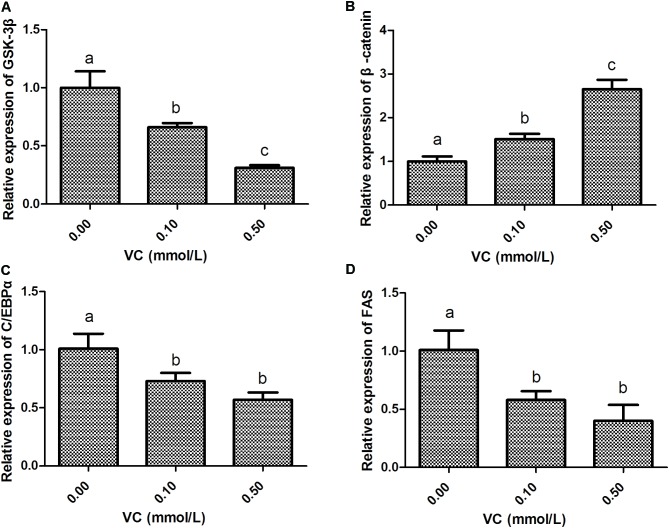
Effect of VC on the mRNA expression in ZF4 cells. **(A)**: GSK-3β; **(B)**: β-catenin; **(C)**: C/EBPα; **(D)**: FAS. Values are expressed as means ± s.e.m. (*n* = 3). Statistically significant differences are denoted by asterisk (*P* < 0.05).

Compared with the control group, the mRNA expression of GSK-3β was significantly decreased (*P* < 0.05), whereas β-catenin expression was significantly induced by 0.1 and 0.5 mmol/L VC treatments (*P* < 0.05) (**Figures 5A,B**). However, the mRNA expression of C/EBPα and FAS in the ZF4 cells of zebrafish was significantly decreased by 0.1 and 0.5 mmol/L VC treatments (*P* < 0.05) (**Figures 5C,D**).

### Effect of VC on FAS Activity and the Content of Glycerol and TG in ZF4 Cells

**FIGURE 6:**
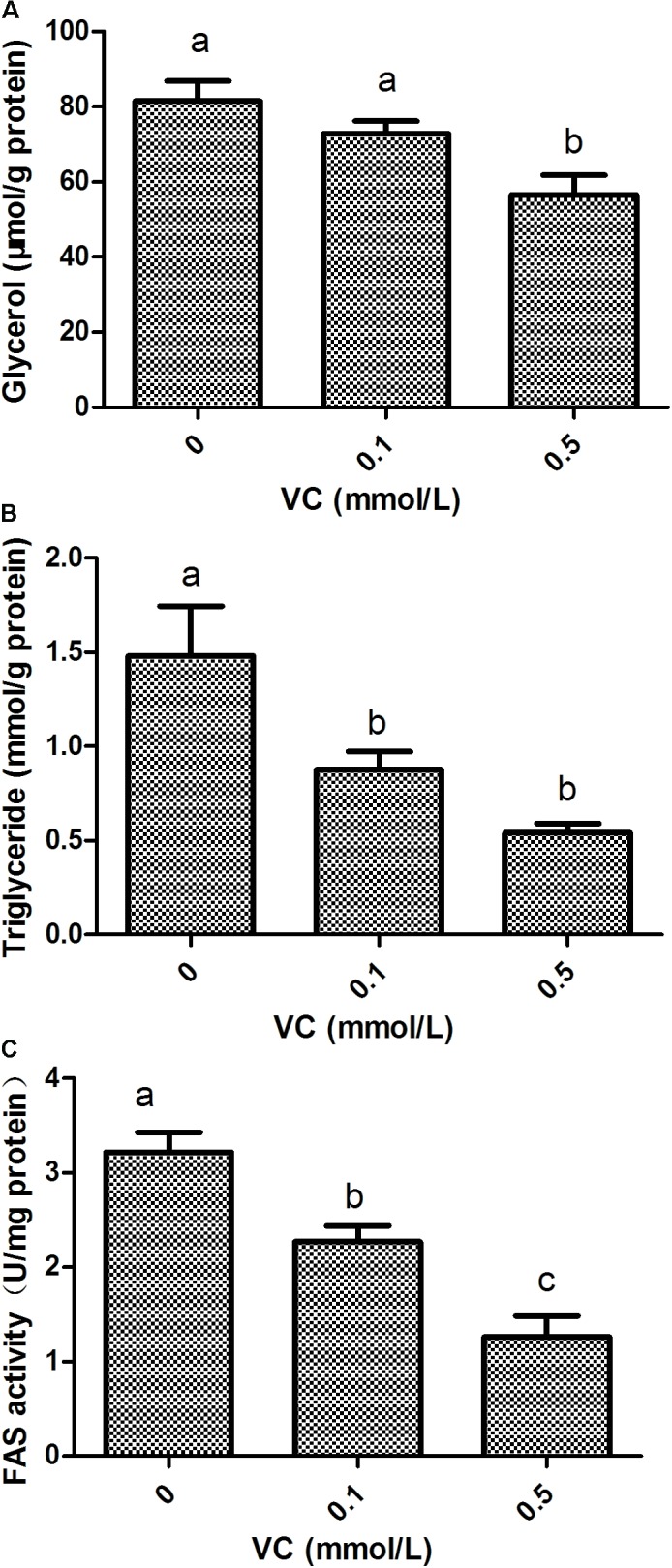
Effect of VC on glycerol, TG, and FAS activity in ZF4 cells. **(A)**: glycerol content; **(B)**: TG content; **(C)**: FAS activity. Values are expressed as means ± s.e.m. (*n* = 3). Statistically significant differences are denoted by different letters (*P* < 0.05).

Compared with the control group, the content of glycerol in ZF4 cells was significantly decreased by 0.5 mmol/L VC treatment (*P* < 0.05) (**Figure 6A**). The content of TG and FAS activity in ZF4 cells was significantly decreased by 0.1 and 0.5 mmol/L VC treatments (*P* < 0.05) (**Figures 6B,C**).

### Effect of VC on the Protein Expression of GSK-3β and β-Catenin in ZF4 Cells

**FIGURE 7:**
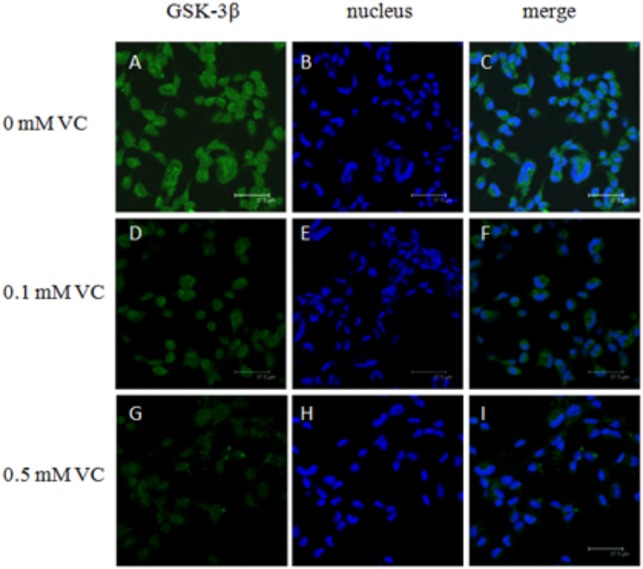
Effect of VC on the immunofluorescence of GSK-3β in the ZF4 cells of zebrafish. **(A,D,G)**: GSK-3β, green; **(B,E,H)**: nucleus, blue; **(C,F,I)**: green and blue merger. Bar: 37.5 μm.

**FIGURE 8:**
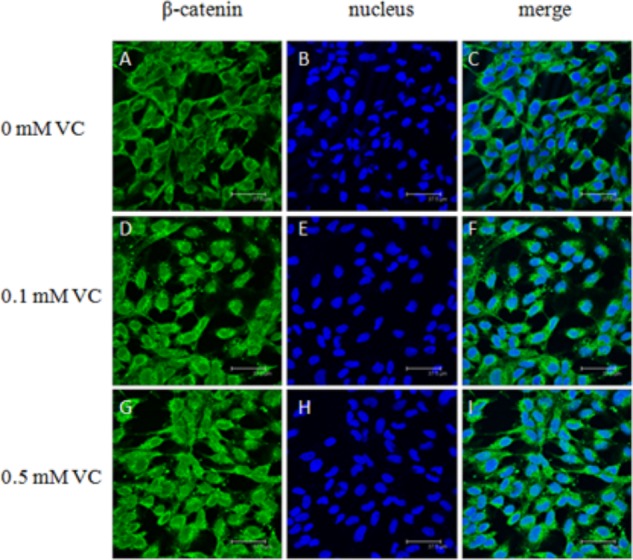
Effect of VC on the immunofluorescence of β-catenin in the ZF4 cells of zebrafish. **(A,D,G)**: β-catenin, green; **(B,E,H)**: nucleus, blue; **(C,F,I)**: green and blue merger. Bar: 37.5 μm.

**FIGURE 9:**
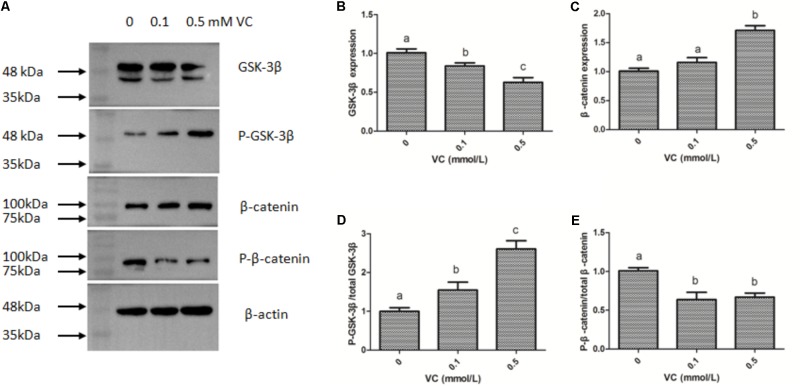
Effect of VC on the protein expression in the ZF4 cells of zebrafish. **(A)** Protein bands of western blot; **(B)** Effect of VC on GSK-3β; **(C)** Effect of VC on β-catenin; **(D)** Effect of VC on the phosphorylation level of GSK-3β; **(E)** Effect of VC on the phosphorylation level of β-catenin. Values are expressed as means ± s.e.m. (*n* = 3). Statistically significant differences are denoted by different letters (*P* < 0.05).

The immunofluorescent assay was carried out to detect the effect of VC on the protein expression of GSK-3β and β-catenin in ZF4 cells (**Figures 7, 8**). It was found that the immunofluorescence of β-catenin was increased, whereas the immunofluorescence of GSK-3β was decreased with increasing VC concentrations (**Figures 7, 8**). The result of western blot indicated that the protein expression level of total GSK-3β was significantly decreased (*P* < 0.05) and that of total β-catenin was significantly increased in ZF4 cells treated with 0.1 and 0.5 mmol/L VC (*P* < 0.05) (**Figures 9A–C**). Moreover, the phosphorylation level of GSK-3β was significantly increased (*P* < 0.05) and that of β-catenin was significantly decreased in ZF4 cells treated with 0.1 and 0.5 mmol/L VC (*P* < 0.05) (**Figures 9A,D,E**).

## Discussion

GSK-3β plays a key role in regulating the canonical Wnt/β-catenin signaling pathway (Dema et al., 2016; Jain et al., 2017). The accumulation of β-catenin leads to the transcription of a genetic program specific for physiological activities (Valenta et al., 2012; Acebron and Niehrs, 2016). In this study, the mRNA expression of GSK-3β was significantly decreased, but the mRNA expression of β-catenin was significantly induced by VC treatments in the muscle and ZF4 cells. The result of western blot indicated that the expression level of total GSK-3β was significantly decreased and that of total β-catenin was significantly increased in ZF4 cells treated with 0.1 and 0.5 mmol/L VC. Moreover, the phosphorylation level of GSK-3β was significantly increased and that of β-catenin was significantly decreased by VC treatments in ZF4 cells. The phosphorylation of β-catenin could be degraded by ubiquitin protease and led to less β-catenin accumulation in the cytoplasm. The decrease of β-catenin phosphorylation level was beneficial to the accumulation of β-catenin. In addition, GSK-3β activity was involved in regulating the phosphorylation of β-catenin, and GSK-3β activity could be inhibited by the increase of GSK-3β phosphorylation and resulted in more β-catenin accumulation in the cytoplasm. The results of gene and protein expression indicated that VC treatments could induce Wnt/β-catenin signaling.

In a previous study, β-catenin signaling suppressed adipogenesis by inhibiting the expression of C/EBPα (Kim et al., 2014; Xu et al., 2014). C/EBPα inhibited adipogenesis by decreasing the expression of FAS in bone marrow (BM)-derived mesenchymal stromal cells and 3T3-L1 preadipocytes (Jang, 2016; Kim and Nam, 2017). Our study also showed that the mRNA expression of C/EBPα and FAS was significantly decreased by VC treatments *in vitro* and *in vivo*. Since β-catenin was activated by VC treatments, β-catenin may further inhibit the expression of C/EBPα and FAS, which was consistent with the previous studies.

It has been found that the dietary VC regulates adipocyte lipolysis, hyperglycemia, and glycosylation in obese diabetic mice (Garcia-diaz et al., 2014; Christie-David et al., 2015; Ellulu et al., 2015; Park et al., 2015; Wilson et al., 2017). Moreover, VC improves the plasma lipid profile (Garcia-diaz et al., 2014; Christie-David et al., 2015; Ellulu et al., 2015; Park et al., 2015; Wilson et al., 2017), and it has been used to treat atherosclerosis by controlling cholesterol levels (Ashor et al., 2014; Zhang et al., 2014; Kobylecki et al., 2015; Polidori et al., 2015). In this study, FAS activity and the content of glycerol and TG in the muscle was significantly decreased by 509.6 and 1007.5 mg/kg VC diets. In ZF4 cells, the content of glycerol was significantly decreased by 0.5 mmol/L VC treatments, and the content of TG and FAS activity was significantly decreased by 0.1 and 0.5 mmol/L VC treatments. Our results indicated that FAS activity and the content of glycerol and TG were related to the concentration of VC. The higher concentration of VC had a better effect on decreasing the content of glycerol and TG.

GSK-3β participated in regulating numerous signaling pathways related to the development of diabetes, neurodegenerative disease, and bipolar disorder (Beurel et al., 2015; Maqbool et al., 2016). Since GSK-3β plays a key role in regulating the canonical Wnt/β-catenin signaling pathway (Dema et al., 2016; Jain et al., 2017), in this study, the gene of GSK-3β was interfered to confirm that GSK-3β/β-catenin was involved in regulating lipid deposition. It was found that the gene expression of β-catenin was increased, whereas the expression of C/EBPα and FAS was decreased by GSK-3β RNA interference. Two-way ANOVA revealed that the factor RNAi had significant effect on FAS activity, glycerol and TG content, as well as the gene expression levels of GSK-3β, β-catenin, C/EBPα, and FAS. However, no significant difference was observed on the factor VC content and the interaction factor in the experiment of GSK-3β RNA interference. For the muscle of zebrafish that was sampled a week after GSK-3β RNA interference, the shorter time with VC treatments may result in no significant difference on the factor VC content. The result of GSK-3β RNA interference indicated that GSK-3β played an important role in lipid-lowering effect by controlling the activity of FAS and the content of glycerol and TG. GSK-3β may be inactivated by VC, which results in β-catenin accumulation, and GSK-3β/β-catenin signaling exerted the lipid-lowering effect by controlling FAS activity and lipid profile in zebrafish.

## Conclusion

The treatment of VC induced the expression of β-catenin by inhibiting GSK-3β expression. GSK-3β/β-catenin signaling exerted the lipid-lowering effect by controlling FAS activity and lipid profile in zebrafish (*Danio rerio*).

## Author Contributions

DL contributed to the conception and design of the work. HY, DL, XZ, and QP contributed to data acquisition, analysis, and interpretation. DL, XZ, and QP drafted the work and revised it critically.

## Conflict of Interest Statement

The authors declare that the research was conducted in the absence of any commercial or financial relationships that could be construed as a potential conflict of interest.
