# Supplementary material for: Investigation of the Lipid-Lowering Effect of Vitamin C Through GSK-3β/β-Catenin Signaling in Zebrafish
Source: Front Physiol. 2018 Aug 14;9:1023. doi: 10.3389/fphys.2018.01023 (PMC6103266; doi:10.3389/fphys.2018.01023)
Supplement: Supplementary file 1 [file Table_1.DOCX]

| **TABLE 1** Effect of RNAi and VC content on the gene expression | | | | | | | |
| --- | --- | --- | --- | --- | --- | --- | --- |
| Gene expression | RNAi | VC content (mg/kg) | | Two-way ANOVA | Factor 1:  RNAi | Factor 2:  VC content | Interaction  Factor 1 x 2 |
|  |  | 8.2 | 1007.5 |  |  |  |  |
| GSK-3β expression | DEPC | 1.00±0.10^a^ | 1.00±0.29^a^ | F | 24.033 | 0.096 | 0.067 |
|  | RNAi | 0.48±0.18^b^ | 0.41±0.17^b^ | P | 0.001 | 0.765 | 0.802 |
|  |  |  |  | Significance | * | ns | ns |
| β-catenin expression | DEPC | 1.00±0.15^a^ | 1.16±0.45^a^ | F | 51.205 | 2.025 | 0.915 |
|  | RNAi | 2.30±0.74^b^ | 3.76±0.69^b^ | P | 0.000 | 0.193 | 0.367 |
|  |  |  |  | Significance | * | ns | ns |
| C/EBPα expression | DEPC | 1.00±0.05^ab^ | 1.27±0.28^a^ | F | 30.134 | 0.681 | 4.286 |
|  | RNAi | 0.69±0.05^bc^ | 0.58±0.14^c^ | P | 0.001 | 0.433 | 0.072 |
|  |  |  |  | Significance | * | ns | ns |
| FAS expression | DEPC | 1.00±0.17^ab^ | 1.09±0.23^a^ | F | 39.019 | 0.771 | 3.062 |
|  | RNAi | 0.58±0.12^bc^ | 0.34±0.09^c^ | P | 0.000 | 0.406 | 0.118 |
|  |  |  |  | Significance | * | ns | ns |

Values are expressed as means ± s.e.m. (*n* = 3). Different letters indicate significant differences (*p* < 0.05) between VC treatments by one-way ANOVA test. Statistically significant differences (two-way ANOVA) between RNAi and VC content are denoted as: ns = not significant, * *p* < 0.05.
