# Supplementary material for: Investigation of the Lipid-Lowering Effect of Vitamin C Through GSK-3β/β-Catenin Signaling in Zebrafish
Source: Front Physiol. 2018 Aug 14;9:1023. doi: 10.3389/fphys.2018.01023 (PMC6103266; doi:10.3389/fphys.2018.01023)
Supplement: Supplementary file 2 [file Table_2.DOCX]

| **TABLE 2** Effect of RNAi and VC content on the biochemical indexes | | | | | | | |
| --- | --- | --- | --- | --- | --- | --- | --- |
| Biochemical index | RNAi | VC content (mg/kg) | | Two-way ANOVA | Factor 1:  RNAi | Factor 2:  VC content | Interaction  Factor 1 x 2 |
|  |  | 8.2 | 1007.5 |  |  |  |  |
| Glycerol content  (μmol/g protein) | DEPC | 88.87±4.15^a^ | 81.19±3.71^ab^ | F | 24.625 | 2.459 | 1.656 |
|  | RNAi | 72.06±5.46^bc^ | 71.30±5.11^c^ | P | 0.001 | 0.155 | 0.234 |
|  |  |  |  | Significance | * | ns | ns |
| TG content  (mmol/g protein) | DEPC | 2.09±0.20^a^ | 1.88±0.05^ab^ | F | 17.092 | 4.912 | 0.262 |
|  | RNAi | 1.73±0.09^b^ | 1.59±0.15^b^ | P | 0.003 | 0.058 | 0.623 |
|  |  |  |  | Significance | * | ns | ns |
| FAS activity  (U/mg protein) | DEPC | 2.52±0.24^a^ | 2.19±0.16^ab^ | F | 26.500 | 5.782 | 0.677 |
|  | RNAi | 1.91±0.17^b^ | 1.74±0.13^b^ | P | 0.001 | 0.043 | 0.434 |
|  |  |  |  | Significance | * | * | ns |

Values are expressed as means ± s.e.m. (*n* = 3). Different letters indicate significant differences (*p* < 0.05) between VC treatments by one-way ANOVA test. Statistically significant differences (two-way ANOVA) between RNAi and VC content are denoted as: ns = not significant, * *p* < 0.05.
